# Proteomic enzyme analysis of the marine fungus *Paradendryphiella salina* reveals alginate lyase as a minimal adaptation strategy for brown algae degradation

**DOI:** 10.1038/s41598-019-48823-9

**Published:** 2019-08-26

**Authors:** Bo Pilgaard, Casper Wilkens, Florian-Alexander Herbst, Marlene Vuillemin, Nanna Rhein-Knudsen, Anne S. Meyer, Lene Lange

**Affiliations:** 1 Enzyme Technology, Department of Biotechnology and Biomedicine, Technical University of Denmark, Lyngby, Denmark; 2 Center for Microbial Communities, Department of Chemistry and Bioscience Aalborg University, Aalborg, Denmark; 3 BioEconomy, Research & Advisory, Copenhagen, Denmark

**Keywords:** Microbiology, Fungi, Proteomics, Molecular ecology

## Abstract

We set out to investigate the genetic adaptations of the marine fungus *Paradendryphiella salina* CBS112865 for degradation of brown macroalgae. We performed whole genome and transcriptome sequencing and shotgun proteomic analysis of the secretome of *P*. *salina* grown on three species of brown algae and under carbon limitation. Genome comparison with closely related terrestrial fungi revealed that *P*. *salina* had a similar but reduced CAZyme profile relative to the terrestrial fungi except for the presence of three putative alginate lyases from Polysaccharide Lyase (PL) family 7 and a putative PL8 with similarity to ascomycete chondroitin AC lyases. Phylogenetic and homology analyses place the PL7 sequences amongst mannuronic acid specific PL7 proteins from marine bacteria. Recombinant expression, purification and characterization of one of the PL7 genes confirmed the specificity. Proteomic analysis of the *P*. *salina* secretome when growing on brown algae, revealed the PL7 and PL8 enzymes abundantly secreted together with enzymes necessary for degradation of laminarin, cellulose, lipids and peptides. Our findings indicate that the basic CAZyme repertoire of saprobic and plant pathogenic ascomycetes, with the addition of PL7 alginate lyases, provide *P*. *salina* with sufficient enzymatic capabilities to degrade several types of brown algae polysaccharides.

## Introduction

Knowledge of the diversity and distribution of marine fungi is increasing in step with improvements in culture-based and molecular identification techniques and increased research efforts^1–9^. Numerous fungal species have been isolated from brown algae^1^ and observations and enzyme screenings have shown them to be able to grow on macroalgal substrates while producing enzymes that catalyze degradation of algal polysaccharides^2–5^, however, only few marine fungi have been genome sequenced and even fewer studied in combination with culture-based analyses of enzymatic degradation of marine macroalgae (seaweeds)^6^.

The observed association of the fungus *Paradendryphiella salina* (Ascomycota) to brown algae dates back to 1916 when the fungus was first described as *Cercospora salina*. Its ecological mode and habitat were described as saprophytic on seaweeds^7^. Several reports investigating enzyme secretion and carbon utilization have confirmed the ability of *P*. *salina* to utilize alginate, laminarin and cellulose from brown algae^8,9^. Based on this we identified *P*. *salina* as an interesting candidate for studying potential adaptations of its enzyme repertoire to the breakdown of brown algae polysaccharides.

Brown macroalgae (Phaeophyceae) are a large and diverse class of marine macrophytes found abundantly in the seas of the southern and northern hemisphere^10^. The cell wall of brown algae consists mainly of alginate, the most abundant polysaccharide found in the outermost cell wall layer^11^, fucose-containing sulfated polysaccharides (FCSPs) and cellulose in a 3:1:1 ratio^10^. Presence of mixed linkage glucan (MLG)^12^ and arabinogalactans linked to proteins have also been reported^13^. Carbon storage in brown algae consists mainly of laminarin, whilst trehalose and mannitol appear to function more as osmotic stress regulators^14^.

As a part of our quest to annotate enzymes for the biorefining of brown algae, this study was undertaken to elucidate the enzymatic repertoire utilized by a known saprophytic marine fungus in the breakdown of brown algae and to examine the degree of enzyme overlap shared by this fungus with its terrestrial counterparts. By using a combination of genomics, culture-based methods and shotgun proteomics we show that *P*. *salina* secretes enzymes capable of catalyzing degradation of a range of polysaccharides found in brown macroalgae, notably also alginate. Our study also indicates that alginate lyase is the only additional enzyme adaptation necessary for allowing *P*. *salina* to degrade brown algae, when compared to its terrestrial relatives.

## Methods

### Brown macroalgae species

*Ascophyllum nodosum* was harvested along the coastline of Norway (European Protein, Bække, Denmark). *Fucus serratus* was harvested in the Kattegat Sea (Dansk TANG, Nykøbing Sj., Denmark). *Saccharina latissima* was harvested along the coastline of the Faroe Islands (Ocean Rainforest, Kaldbak, Faroe Islands). All three species were obtained in dry form and milled to particle sizes under 1 mm.

### Fungal strain and culture conditions

*Paradendryphiella salina* CBS112865 (G.K. Sutherland) Woudenb. & Crous, isolated from *F*. *serratus* in the North Sea, was obtained from CBS-KNAW culture collection (www.westerdijkinstitute.nl/collections/) and maintained on 2% oatmeal agar. Spores were added in a final concentration of 10^5^ spores/ml. The base medium used for all fermentations was prepared with 0.10% (w/v) glucose and otherwise as previously described^15^. The three species of powdered brown algae were autoclaved (121 °C, 20 min.) before being added to the base medium at 2% w/v. The carbon limited medium consisted solely of the base medium. All fungal fermentations were carried out in five replicates (23 °C, 180 rpm, 14 days) in standard 250 ml Erlenmeyer flasks. All fermentations were routinely checked for contamination by microscopy. One of the replicates in the *P*. *salina* fermentation of *A*. *nodosum* failed due to an inoculation error, thus only four yielded results.

### Genome and transcriptome sequencing and assembly

DNA was extracted from *P*. *salina* mycelium growing in liquid YPD medium for 4 days using a Qiagen (Venlo, Holland) DNeasy plant minikit. RNA was extracted from mycelium growing in liquid base medium with 2% *S*. *latissima* for 7 days using a Qiagen RNeasy minikit. mRNA was purified by polyA capture, fragmented and converted to double-stranded cDNA. The “dUTP method” was used to generate strand-specific mRNA-seq libraries^16^. Paired-end sequence reads (125 bp) were generated using an Illumina HiSeq 2500. FASTQ sequence files were generated using Illumina Casava (1.8.3) for DNA and bcl2fastq2 (2.18) for RNA. The draft genome was assembled using the CLC genomics workbench (9.5.1). Misassemblies and nucleotide disagreement between the Illumina data and the contig sequences were corrected with Pilon^17^ (1.20). The contigs were linked into scaffolds using SSPACE Premium scaffolder (2.3)^18^ and the gapped regions within the scaffolds were partially closed in an automated manner using GapFiller (1.10)^19^. The transcriptome was assembled by performing genome alignment using Tophat2 (2.1.1)^20^. The alignment was used to guide a de novo assembly of the RNA reads using Trinity (2.4.0)^21^.

### Gene prediction and annotation

Genome-wide gene prediction was accomplished by creating a training set using AUGUSTUS (2.7) (http://bioinf.uni-greifswald.de/webaugustus/)^22^ using the assembled transcripts as hints. The predicted protein sequences were annotated using HMMer3^23^ with dbCAN^24^ models using an E-value threshold of 10^−5^, InterProScan 5^25^ and BLASTp^26^ (https://blast.ncbi.nlm.nih.gov/Blast.cgi). Phobius^27^ and Euk-mPLoc (2.0)^28^ (http://www.csbio.sjtu.edu.cn/bioinf/euk-multi-2/) were used for subcellular localization and transmembrane domain prediction. For functional predictions of CAZymes, HotPep^29^ and CUPP^30^ were used. Substrate specificities of CAZymes were inferred by manual inspection of CAZy (www.cazy.org)^31^ and the BRENDA database (www.brenda-enzymes.org)^32^. Another version of HotPep was used for prediction of protease families^33^ (https://sourceforge.net/projects/hotpep-protease/). For all accepted annotations at family level, at least two different methods had to agree. Sulfatase annotation consisted of BLASTp searching sulfatase hits found with InterProScan in the SulfAtlas^34^ database (http://abims.sb-roscoff.fr/sulfatlas/) with E-value 0 as threshold.

### Phylogenetic analysis

Phylogenetic analyses were performed by aligning the amino acid sequences of the putative catalytic domains with Mafft^35^ which were manually inspected in CLC main workbench (8). Maximum likelihood analyses were performed with RaxML blackbox^36^ using WAG as substitution matrix and otherwise default parameters at the CIPRESS server (www.phylo.org/)^37^. RaxML reached the MRE-based Bootstopping criterion after 252 replicates.

### Genome comparison

Genome protein sequences were downloaded from Genbank^38^. Local BLASTp searches of protein sequences were executed in CLC main workbench (version 8) with default parameters. Venn analysis and diagram development of CAZymes were performed online (http://bioinformatics.psb.ugent.be/webtools/Venn/).

### Carbohydrate monomer composition

Carbohydrate monomer composition was determined by HPAEC-PAD analysis following a two-step sulfuric acid hydrolysis as previously described^39^.

### Shotgun proteomic analysis of fungal secretomes

Protein precipitation was performed as previously described^40^. The precipitated proteins were diluted in digestion buffer, consisting of 10% Acetonitrile and 50 mM HEPES buffer pH 8.5. 10 μg of protein from each sample was reduced, alkylated and in-solution digested with trypsin (Sigma-Aldrich, St. Louise, MI, US) and LysC (Wako, Osaka, Japan) and desalted on C18 filters (Thermo Fisher Scientific, Rockford, USA). 1 μg from each sample was analysed by Liquid Chromatography-tandem Mass Spectrometry (LC-MS/MS) as previously described^40^. Protein identification was performed using the open-source software MaxQuant (1.6.3.4)^41^ and Perseus^42^ as previously described^40^. A hit was only considered if present in three out of five biological replicates. The iBAQ values were normalized across the samples by multiplying them with factors calculated from the original protein concentration in the supernatants (Supplementary Table S1) before being averaged across the replicates and used for further analyses.

### Enzyme assays of supernatants

The AZurine CrossLinked (AZCL) assay was carried out in 96-well microtiter plates as suggested by the manufactor. Each of the 12 selected AZCL substrates (Supplementary Table S2) (Megazyme, Bray, Ireland) was prepared by adding 0.01% w/v of substrate to 0.1% agarose dissolved in 0.05 M Britten-Robinson (BR) buffer^43^ (pH 5). Each well contained 200 μl of substrate and 100 μl of supernatant. The plates were incubated (30 °C, 45 h), centrifuged (15 min. 3000 *g*) and 100 μl of reaction was transferred to a new plate and quantified by measuring A_590_.

Alginate lyase activity was assayed on 1.5 mg/ml sodium alginate from *F*. *vesiculosus* (Sigma) mixed with 10 mM BR buffer (pH 5). The reactions were carried out in 96-well microtiter plates by mixing 20 μl of sample with 180 μl of substrate and incubating (30 °C, 120 min.) Degradation of substrates was monitored by measuring A_235_.

Fucoidanase activity was assayed on fucoidan extracted from *Fucus evanescens* and *Fucus vesiculosus* using Carbohydrate–Polyacrylamide Gel Electrophoresis (C-PAGE) as previously described^44^.

### Genes, cloning, expression and purification of PL7 alginate lyase

An open reading frame encoding PsAlg7A (ENA acc. LR536815) was identified in *P*. *salina*. The gene sequence was truncated to exclude the 22 amino acids long predicted signal peptide. The codon optimized gene including a C-terminal His-tag (Supplementary Table S3) for *Pichia pastoris* was cloned into pPICZαA (GenScript, Piscataway, NJ, USA). The resulting construct was transformed into *E*. *coli* strain DH5α and selected on low salt LB agar plates with 25 μg ml^−1^ zeocin and propagated in low salt LB medium with 25 μg ml^−1^ zeocin (Invitrogen, Carlsbad, CA, USA). The pPICZαA-PsAgl7A construct was linearized by PmeI (New England BioLabs, Ipswich, MA, USA), transformed into *P*. *pastoris* X-33 by electroporation (Micropulser; Bio-Rad, Hercules, CA, USA), and selected (30 °C, 3 days) on yeast peptone dextrose plates with 100 μg ml^−1^ zeocin. Transformants were grown in a 5 L Sartorius Biostat Aplus fermenter via glycerol fed batch fermentation at 30 °C for 24 hours. Then, a methanol fed-batch phase was initiated to induce expression and secretion of the enzyme. After 96 hours of methanol induction at 20 °C. The cell-free supernatant was concentrated and buffer-exchanged into 50 mM Tris, 500 mM NaCl, 20 mM imidazole pH 7.5 by ultrafiltration using the cross-flow filter reactor equipped with a 10 kDa cutoff membrane (Millipore, Sartorius, Goettingen, Germany). The supernatant was applied to a 5 ml HisTrap FF HP column (GE Healthcare Uppsala, Sweden) equilibrated with 50 mM Tris, 500 mM NaCl, 20 mM imidazole pH 7.5 and eluted (2 ml.min^−1^) by a linear 20–500 mM imidazole gradient (30 CV). Fractions containing the target enzymes were pooled, concentrated (Viaspin (10 kDa), Goettingen, Germany), Sartorius) and applied to a Hiload 16/60 Superdex G75 column (GE Healthcare) equilibrated with 10 mM NaOAc, 150 mM NaCl, pH 6 (0.5 ml.min^−1^). Fractions containing pure target enzymes were concentrated (Viaspin (10 kDa), Sartorius) and stored at 4 °C. The purity was checked on 12% SDS-PAGE gels. The theoretical molar extinction coefficient and size were calculated using ProtParam (http://web.expasy.org/protparam) to 30035 M^−1^ cm^−1^ and 25.445 kDa. Protein concentrations were determined by A_280_ using the theoretically obtained molar extinction coefficients.

### Enzyme kinetics assays

Reactions on 0.1875 mg ml^−1^ of PsAlg7A were set up in triplicates in a 96 well quartz plate. Alginate (Sigma), polyguluronic acid (larger than 5 kDa) (Carbosynth, Compton, UK), and polymannuronic acid (larger than 5 kDa) (Carbosynth) substrates were prepared in 20 mM Britten-Robinson, 200 mM NaCl, pH 5 buffer. Substrate concentrations ranging from 0.025 to 1.5 mg ml^−1^ were assayed. To determine enzyme kinetics, the averaged initial velocities (linear range over 10 minute runs) in milli-absorbance units (mAU) at A_235_ were calculated to mM per seconds of 4-deoxy-4,5-unsaturated mono-uronates from measurement of double bonds at absorbance of 235 nm caused by lyase induced β-elimination, versus the substrate concentrations using the extinction coefficient of 6150 M^−1^ cm^−1^^45,46^.

## Results

### Genome properties and comparison

The draft genome of *P*. *salina* was assembled to a size of 27.4Mbp with an average GC content of 52%, a N50 of 32 K and an average scaffold size of 15062 bp. The assembled transcriptome contained 21394 transcripts with an average size of 1789bp and a N50 of 2792 (Supplementary Table S4). For gene prediction both the genome and transcripts were submitted to the AUGUSTUS server, which predicted 9281 proteins in the genome (Supplementary File S1). The transcripts were used as is, in a tBLASTn search towards the genome predicted proteins and were found to match approximately 8600 of predicted proteins after filtering (identity >75%, HSP length >200 bp) (Supplementary File S2). The combined annotations from CUPP, HotPep, dbCAN and InterProScan predicted putative domains for 8184 proteins of which 448 contained CAZyme domains. CUPP and HotPep annotated putative functions for 323 of the putative CAZymes (Supplementary File S2).

**Figure 1:**
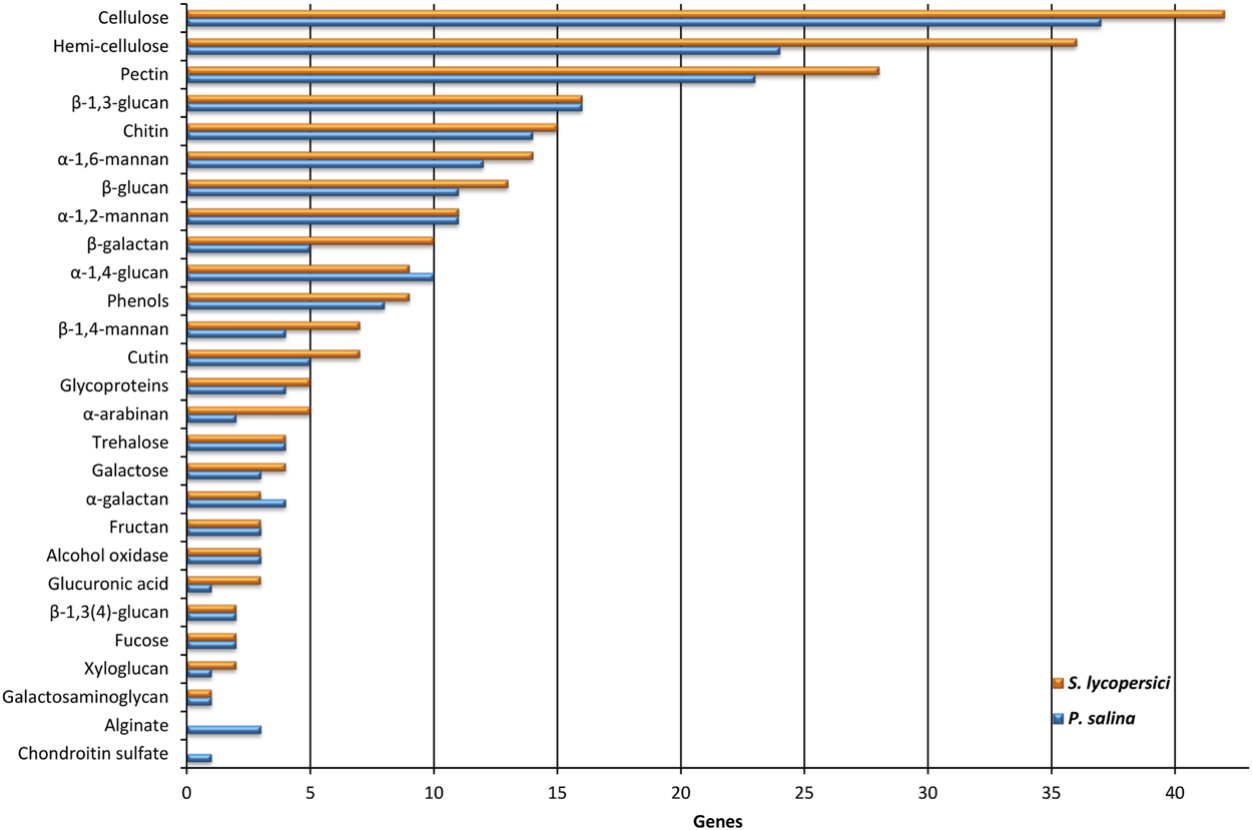
Comparative distribution of CAZymes in *P*. *salina* and its terrestrial relative *S*. *lycopersici*, binned according to substrate specificities. CAZy domains were annotated with dbCAN HMM models and CUPP. Substrate designations were inferred by using the predicted functions from CUPP (Supplementary Files S2 and S3).

In order to elucidate *P*. *salina* potential genetic macroalgae specific specializations, a genome comparison of a terrestrial fungus belonging to the same family was undertaken. Candidates were found by BLASTn (megablast)^47^ of the ribosomal barcode from *P*. *salina* (accession MH873443.1) against the The Whole Genome (WGS) blast database with restriction to Pleosporaceae (taxid:28556). The first hit was the terrestrial plant pathogen *Stemphylium lycopersici* at E-value 0.0, 100% query coverage and 98% identity (Supplementary Table S5). The result correlated with the reported close taxonomic relationship of *P*. *salina* and *Stemphylium* species^48^. The protein sequences from the *S*. *lycopersici* genome (accession ASM119154v1)^49^ were subjected to the same annotation analyses as *P*. *salina* and used for comparisons (Supplementary File S3). The *P*. *salina* and *S*. *lycopersici* cross genome BLASTp showed a 98.3% agreement between the annotations of CAZymes and an average pairwise identity of 88% (±10). Of the 9282 predicted *P*. *salina* proteins, 7581 showed 75% identity or higher to *S*. *lycopersici* proteins, 461 proteins fell under the E-value threshold of these, and 274 remained unannotated (Supplementary File S3). The CAZyme annotations were binned according to broad substrate specificities and compared. The two fungi showed similar substrate profiles, however, *P*. *salina* generally had reduced numbers of CAZYme encoding genes in most categories related to polysaccharides found mainly in the primary plant-cell walls of terrestrial plants, the most significant being hemicellulose, pectin, β-galactan and α-arabinan. Chondroitin AC lyase (chondroitin sulfate) and alginate lyase were unique to *P*. *salina* (Fig. 1).

**Figure 2:**
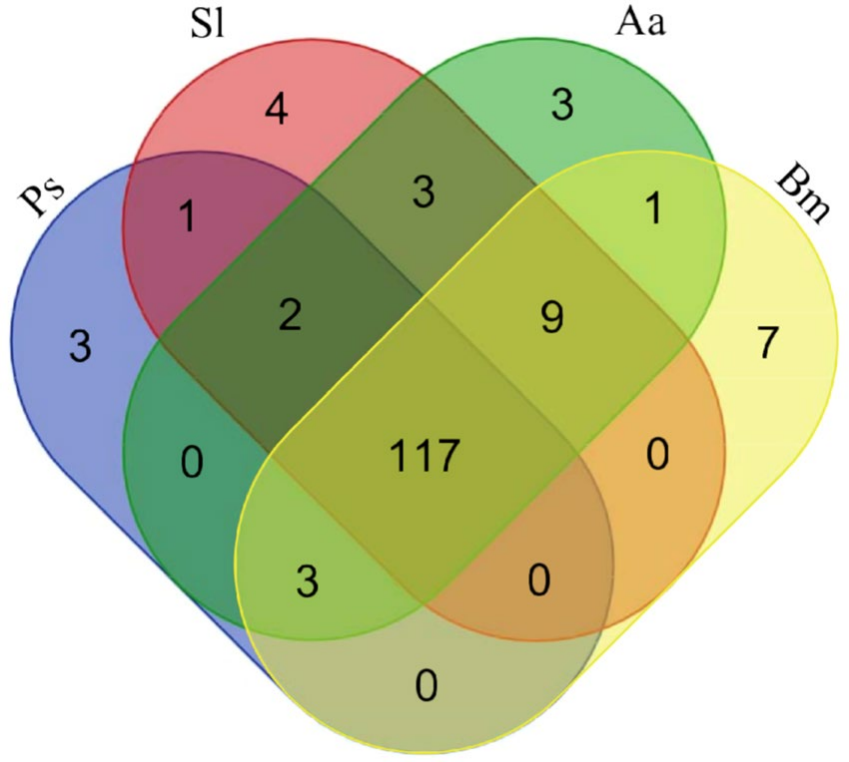
Venn diagram of shared CAZyme domain families between *P*. *salina* (Ps), and the terrestrial fungi: *S*. *lycopersici* (Sl), *A*. *alternata* (Aa) and *B*. *maydis* (Bm). The domains were annotated using dbCAN HMM models and CUPP (Supplementary Table S6). The three unique families in *P*. *salina* (indicated in the blue zone) were PL7, PL8 and CBM24.

Protein sequences from two additional fungal plant pathogens, *Bipolaris maydis* (Ascomycota) (accession GCF_000354255.1)^50^ and *Alternaria alternate* (Ascomycota) (accession GCF_001642055.1)^51^, found amongst the top BLASTn results were included in a broad CAZyme comparison, to broaden the taxonomic variance. The CAZyme profiles showed high similarity with respect to families and paralogue copies within them (Fig. 2) (Supplementary Table S6). Compared to the three terrestrial fungal plant pathogens, *P*. *salina* harbored 25% fewer CAZyme domains than *S*. *lycopersici* and *B*. *maydis* and 35% fewer than *A*. *alternata*. Approximately the same relationship applied for the putative sugar transporters (Table 1).

**Table 1:** Summary of annotated CAZymes and potential sugar transporters in *P*. *salina* (Ps), *S*. *lycopersici* (Sl), *B*. *maydis* (Bm) and *A*. *alternata* (Aa).

Organism	Auxiliary Activities	Carbohydrate Esterases	Polysaccharide Lyases	Glycosyl- Transferases	Glycoside Hydrolases	Total CAZymes	Major Facilitator (PF07690)	Sugar (and other) transporter (PF00083)	Total transporters
Ps	86	55	22	209	82	454	157	73	230
Sl	136	80	23	278	99	616	205	91	296
Bm	141	77	16	274	99	607	219	78	297
Aa	167	95	26	303	108	699	256	104	360

CAZymes were annotated using dbCAN HMM models and CUPP and putative transporter genes were annotated using PFAM HMM models.

Unique to *P*. *salina* were three PL7 alginate lyases, in addition to a PL8 protein with similarity to ascomycete chondroitin AC lyases, and a CBM24 appended to a GH18 domain (Fig. 2). Family GH18 harbors enzymes such as chitinases and CBM24 has been reported to bind to α-1,3-mutan, a mixed linkage glucan from *Streptococcus sp*.^52^.

### Brown algae sugar monomer composition

**Figure 3:**
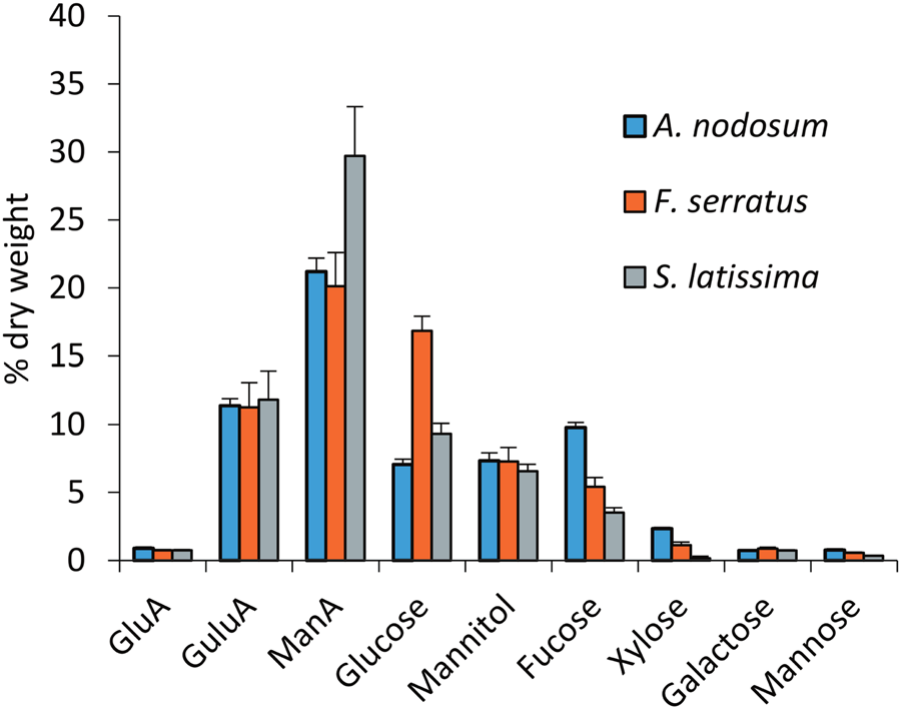
HPAEC-PAD based analysis of monomer sugars in three species of brown algae. GluA (glucuronic acid), GuluA (guluronic acid), ManA (mannuronic acid). The samples were analyzed in triplicates.

Three brown algae species *A*. *nodosum*, *F*. *serratus* and *S*. *latissima* were selected representatives of the orders Fucales and Laminariales, commonly found in the North Sea. To assess the content and variation of sugars in the selected species, we performed monomer composition analysis by acid hydrolysis with subsequent HPAEC-PAD of products. The analysis showed the combined content of guluronic and mannuronic acids, which constitute alginate, to be as high as 30–40% in all three species of algae (Fig. 3). Fucose content, assumed to mainly stem from FCSPs, varied significantly across the three species, with *A*. *nodosum* containing almost 10%. The majority of the glucose is assumed to come from laminarin and cellulose of which *F*. *serratus* contained almost double the amount compared to *A*. *nodosum*. Mannitol content was relatively the same for all the species at approximately seven percent. The total sugar content averaged around 62% of the dry weight (Fig. 3).

### Enzymes relevant for brown algae cell wall degradation in the *P*. *salina* genome

Based on the combination of assumed substrate specificities of the CAZymes and the monomer analysis of the brown algae, it was possible to identify enzymes in the *P*. *salina* genome relevant for the degradation of all major types of polysaccharides in brown algae except FCSPs (Table 2).

**Table 2:** Overview of putative CAZymes found in the *P*. *salina* genome relevant for degrading polysaccharides in brown algae. Family and functional annotation was performed using dbCAN and CUPP (Supplementary File S2).

Substrate	Enzyme activity	EC no.	CAZyme families				
Alginate	Mannuronate-specific alginate lyase	4.2.2.3	3 PL7				
Cellulose	Lytic Polysaccharide MonoOxygenase (LPMO)	—	25 AA9				
	Cellobiose dehydrogenase (acceptor)	1.1.99.18	2 AA3				
	Endo-β-1,4-glucanase	3.2.1.4	2 GH5	2 GH45	1 GH12		
	β-1,4-cellobiohydrolase (reducing end)	3.2.1.176	3 GH7				
	β-1,4-cellobiohydrolase (non-reducing end)	3.2.1.91	2 GH6				
	β-glucosidase	3.2.1.21	3 GH1	9 GH3			
β-1,3-glucan	Endo-β-1,3-glucosidase	3.2.1.39	4 GH16	2 GH17	1 GH64	2 GH81	3 GH128
	Exo-β-1,3-glucanase	3.2.1.58	4 GH5	3 GH55			
	Endo-β-1,6-glucanase	3.2.1.75	2 GH5				
	Exo-β-1,3/1,6-glucanase	3.2.1.-	2 GH131				
	β-glucosidase	3.2.1.21	3 GH1	9 GH3			
	β-1,3-glucosidase	3.2.1.-	1 GH132				
Mixed linkage glucan	Endo-β-1,3(4)-glucanase	3.2.1.6	2 GH16				
	Endo-β-1,3-glucosidase	3.2.1.39	2 GH17				
Arabinogalactan	β-1,3-galactosidase	3.2.1.145	1 GH43				
	Endo-β-1,6-galactosidase	3.2.1.164	2 GH5				
	β-galactosidase	3.2.1.23	3 GH2	2 GH35			
Trehalose	α,α-trehalase	3.2.1.28	2 GH37	1 GH65			

**Figure 4:**
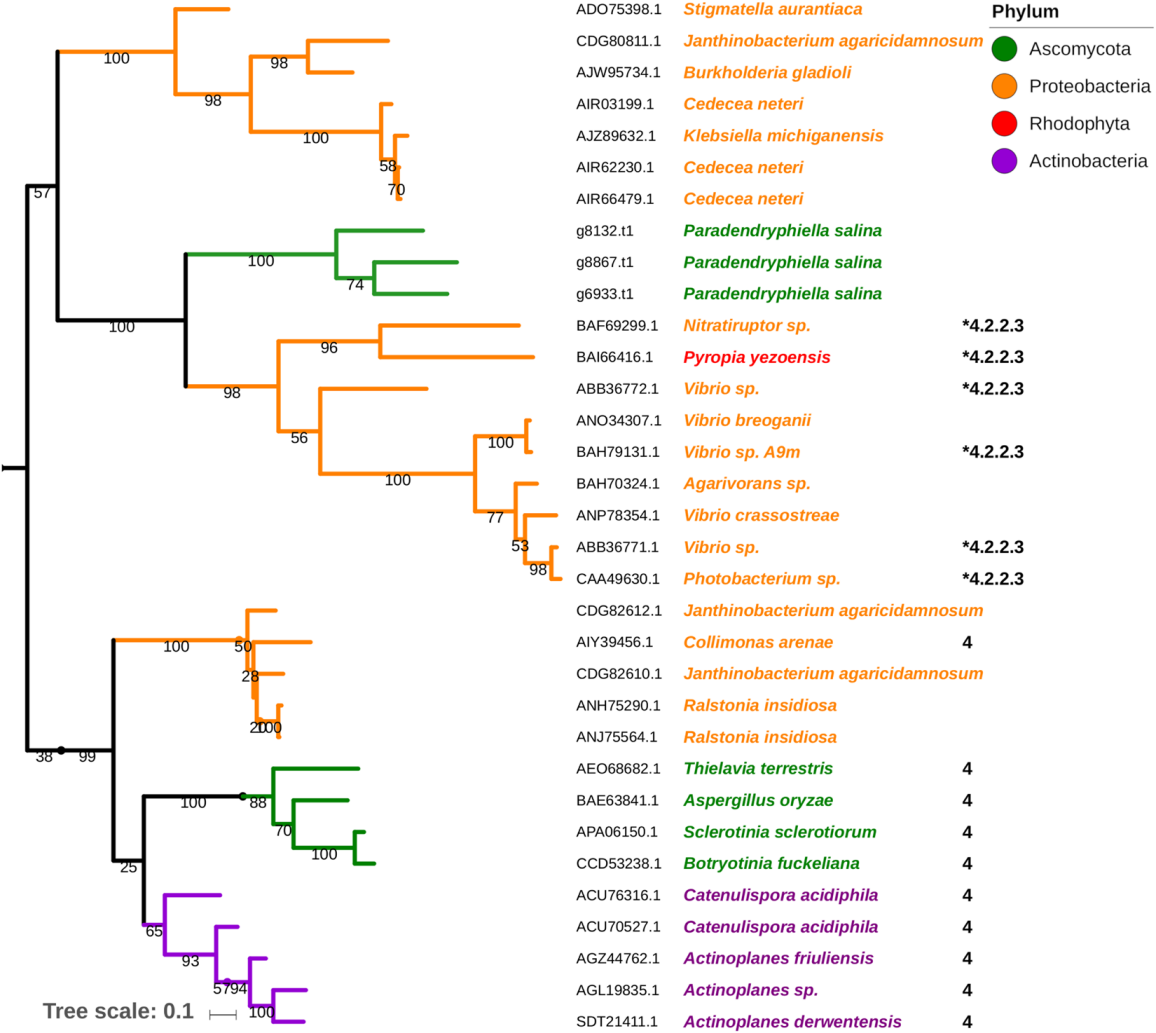
Maximum likelihood phylogenetic analysis of selected CAZy-listed PL7 protein sequences, including the three sequences from *P*. *salina* found in this study. The species names are indicated along with accession numbers of corresponding PL7 sequences. Branch numbers indicate bootstrap values. The tree scale bar indicates substitution changes per site. A *indicate EC numbers of characterized members listed in CAZy. Sequences belonging to PL7 subfamily 4 are indicated in the same column. The tree was pruned from a larger phylogenetic tree containing all CAZy listed sequences (Supplementary Fig. S1).

The predicted PL7 genes were found on three different contigs and interestingly none of them contained introns. The GC percentage did not significantly vary from the contigs they were found in or the average GC content of the genome (Supplementary File S4). The top ten hits of the BLASTp analysis revealed marine bacterial sequences with the highest identities at a maximum of 30% similarity and no fungal hits despite several fungal sequences being listed in CAZy (Supplementary Table S7). Furthermore, the fungal PL7 members in CAZy all belong to subfamily four, but the dbCAN result of the three *P*. *salina* sequences yielded no such hit (Supplementary File S2). To expand on this, the sequences were aligned with PL7 domain sequences derived from CAZy and analyzed in a maximum likelihood phylogenetic analysis (Fig. 4 and Supplementary Fig. S1). The three *P*. *salina* PL7 sequences clustered together in a clade dominated by marine proteobacteria and a single sequence from the red seaweed *Pyropia yezoensis*, all without any subfamily classifications. All characterized members of this clade have been characterized as β-mannuronate specific lyases^53–56^ (EC 4.2.2.3) (Fig. 4). Recently three putative PL7 proteins were annotated in a marine *Calcarisporium* sp. KF525 (Basidiomycota)^57^ and it would have been interesting to include these sequences in the phylogenetic analysis, however no sequence information from this genome was available at the time of this study.

We found putative genes for the complete degradation of cellulose in *P*. *salina* (Table 2). Additionally, a total number of 25 putative AA9 Lytic Polysaccharide MonoOxygenases (LPMOs) genes were found. The AA9 LPMOs have been reported to act mainly on cellulose which is the broad category chosen for them in this study, but there have also been reports of specificity towards hemicellulose^58^ and xyloglucan^59^.

Three different sulfatases were annotated: an aryl S1_6 N-acetylglucosamine-6-sulfatase (EC 3.1.6.14) (g8134.t1), a S1_12 choline-sulfatase (EC 3.1.6.6) (g7726.t1) and a S3 alkyl sulfatase (g9014.t1). Only the S1_6 sulfatase of these three was predicted for extracellular secretion. Five sulfate transporter proteins (PF00916, PF01740) were also found: one sulfate permease 1 (2.A.53.1.1) (g6037.t1), three sulfate permease 2 proteins (2.A.53.1.2) (g1349.t1, g2846.t1, g5922.t1) and a putative sulfate transporter (2.A.53.1.11) (g1670.t1) (Supplementary File S2).

### The *P*. *salina* genome contains enzymes relevant for degradation of algal carbon storage compounds

A wide variety of putative β-1,3-glucanases were identified in the *P*. *salina* genome (Table 2). It is also likely that some of the numerous putative GH1 and GH3 β-glucosidases identified are active on β-1,3 oligosaccharides catalyzing the release of glucose.

We found several enzymes belonging to the mannitol II degradation pathway. A mannitol dehydrogenase (EC 1.1.1.255) (g460.t1), which converts d-mannitol into d-mannose, which is further converted to d-mannose-6-phosphate by hexokinases and then to d-fructose-6-phosphate by mannose-6-phosphate-isomerase before entering the glycolysis II pathway^60^. Two such putative isomerases and multiple hexokinases were found. The presence of one mannitol-1-phosphate 5-dehydrogenase (EC 1.1.1.17) (g1637.t1) belonging to the mannitol I degradation pathway indicates *P*. *salina* has its own mannitol metabolism^61^ (Supplementary File S2).

Three putative trehalase genes (EC 3.2.1.28) were found in *P*. *salina*: one GH65 with a CBM32 and a signal peptide appended and two GH37, the latter also containing a signal peptide but no CBM (Table 2). The presence of three GT20 genes, two α, α-trehalose-phosphate synthases (EC 2.4.1.15) and one trehalose-phosphatase (EC 3.1.3.12) (Supplementary File S2) indicates that *P*. *salina* also has its own trehalose pathway.

### Oxidative enzymes

A total of 16 putative peroxidase genes were found: four AA2 peroxidases, five catalases, three haem peroxidases, one glutathione peroxidase, two chloroperoxidases and a bromoperoxidase/chloroperoxidase gene (Supplementary File S2). In a comprehensive search for vanadium-dependent haloperoxidases (VHPO’s), the genome was searched for all PAP2 domains (PF01569). Nine sequences were found which were subsequently aligned with the sequences used by Fournier and colleagues^62^ and analyzed with maximum likelihood analysis. The phylogenetic tree showed that one PAP2 sequence (g2873.t1) was positioned with vanadium-dependent bromoperoxidases (VBPOs) from brown algae and another sequence along with vanadium-dependent chloroperoxidases from fungi (VCPOs) (g3610.t1). The remaining PAP2 sequences were positioned with putative acid phosphatases (Supplementary Fig. S2).

Other putative oxidative enzymes found included eight laccases, numerous AA7 sugar oxidases, AA3 oxidoreductases and five tannases (Supplementary File S2).

### *P*. *salina* produces active enzymes under both carbon limitation and fermentation of brown algae

**Figure 5:**
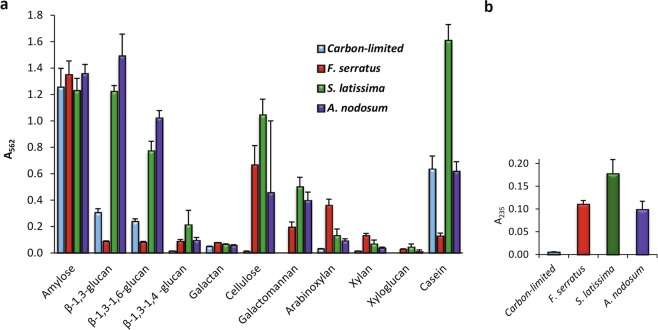
Enzyme activities in the fermentation supernatants. The supernatants of *P*. *salina* after 14 days of incubation on brown algae and under carbon starvation were assayed on a variety of substrates at pH 5 and 30 °C. (**a**) AZCL assay of endo-lytic activities after 45 hours of incubation. (**b**) Alginate lyase activity after two hours of incubation. Five biological replicates for each type of fermentation were analyzed.

We tested the fermentation supernatants on a variety of substrates found in plant, fungal and brown algal cell walls. Overall α-amylase activity was equally strong in all fermentations regardless of substrate (Fig. 5a). In the carbon limited fermentation, amylase activity was observed very soon after day two together with protease activity (Supplementary Fig. S3). Endo-β-1,3-glucanase activity was also observed in all fermentations, but at significantly higher levels in two of the brown algae fermentations (Fig. 5a). Unique to the brown algae fermentations were endo-β-1,4-glucanase, endo-β-1,4-mannanase, endo-1,4-xylanase, mixed linkage endo-β-glucanase (Fig. 5a) and alginate lyase activity (Fig. 5b). No fucoidanase activities were observed in the C-PAGE (Supplementary Fig. S4).

### Characterization of PsAlg7A confirmed the preference for mannuronic acid

**Figure 6:**
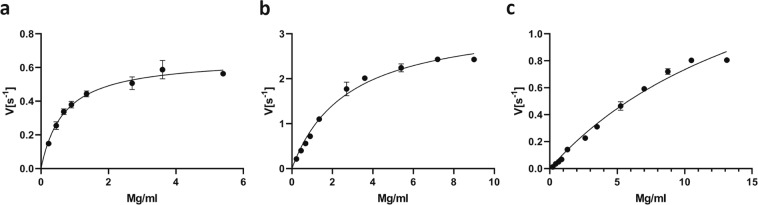
Michaelis-Menten plots of PsAlg7A. Evolution of initial velocities measured as the formation of double bonds for increasing substrate concentrations of (**a**) alginate, (**b**) polymannuronic acid and (**c**) polyguluronic acid.

To confirm the in silico prediction of the mannuronic acid specificity (EC 4.2.2.3) of the PL7 lyases we chose the most abundantly secreted PL7 (PsAlg7A) (g8132.t1) for recombinant expression in *P*. *pastoris*. The recombinant PsAlg7A had an approx. size of 25 kDa according to the SDS-PAGE (Supplementary Fig. S5, lane 2), which corresponds with the predicted molecular mass. PsAlg7A displayed a higher *k*_cat_ for polymannuronic acid than for alginate or polyguluronic acid and a lower *K*_m_ for polymannuronic acid compared to polyguluronic acid (Table 3, Fig. 6), confirming the in silico predicted specificity for PsAlg7A.

**Table 3:** PsAlg7A kinetic parameters on alginate, polymannuronic acid and polyguluronic acid. Values ± denotes the standard error.

Substrate	V_max_ (μM/s)	K_m_ (mM)	K_cat_ (s^−1^)	K_cat_/K_m_ (mM^−1^ s^−1^)
Alginate	0.49 ± 0.03	0.68 ± 0.12	0.66 ± 0.04	0.97
polymannuronic acid	2.5 ± 0.15	2.8 ± 0.41	3.4 ± 0.18	1.2
polyguluronic acid	1.9 ± 0.38	19 ± 5.4	2.1 ± 0.33	0.11

### The secreted proteome of *P*. *salina* reveal similarities and differences in enzyme profiles depending on which algal strain is used as substrate

Maxquant identified 662 proteins from *P*. *salina* across the fermentations (Supplementary File S2).

**Figure 7:**
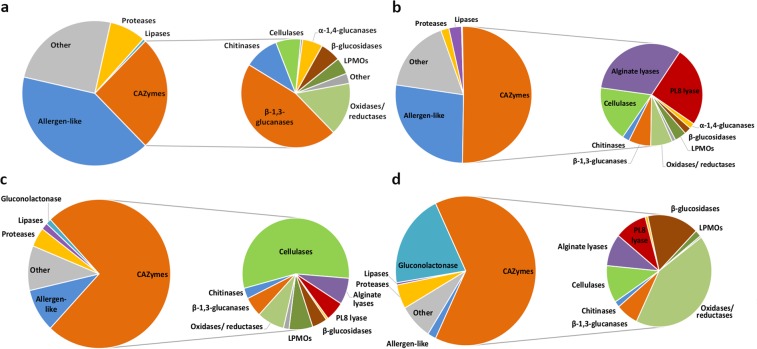
Relative composition of the secreted proteomes of *P*. *salina* incubated on three species of brown algae and under carbon starvation. (**a**) Carbon limited, (**b**) *F*. *serratus*, (**c**) *S*. *latissima*, (**d**) *A*. *nodosum*. The compositions were derived from LC-MS/MS calculated iBAQ values (Supplementary File S2). Proteins representing less than one percent were accumulated under the “other” category.

The relative quantity analysis of the secretomes of the four fermentations revealed different profiles for each of the four types (Fig. 7). As expected, based on the enzyme activities, CAZymes were represented two to three times higher in the fermentations with algae, both in relative distribution (Fig. 6b–d), but also in quantities (Supplementary File S2) compared to the carbon limited fermentation which favored proteases and other proteins (Fig. 7a).

Highly abundant in all fermentations were several α-glucosidases (EC 3.2.1.20), a starch specific AA13 LPMO with an appended CBM20 and a GH13 α-amylase (EC 3.2.1.1) also with a CBM20 appended; the latter enzyme was most likely constitutively expressed and responsible for the observed amylase activity (Fig. 5a). Starch-like polysaccharides or the genes necessary for their synthesis have not been found in brown algae.

The fermentations with algae contained significantly higher levels of PL7 alginate lyases (5–16%), as was expected based on the observed alginate lyase activity (Fig. 5b), but surprisingly also of the PL8 lyase that constituted 4–12% of the observed proteins in the fermentations with algae (Fig. 7).

All three algal *P*. *salina* fermentations contained high amounts of cellulases, in particular high in the *S*. *latissima* fermentation where cellulases constituted 41% of the total protein content (Fig. 7d). This was due to two reducing end acting GH7 β-1,4-cellobiohydrolases (EC 3.2.1.176) (g4260.t1, g793.t1) that constituted approximately 35% of the proteome. Together with a non-reducing end-acting GH6 β-1,4-cellobiohydrolase (EC 3.2.1.91) (g468.t1) and a GH5 endo-1,4-β-glucanase (EC 3.2.1.4) (g5427.t1) (Supplementary File S2), these were most likely linked to the observed cellulase activity (Fig. 5a).

The majority of CAZymes in the carbon limited fermentation consisted of β-1,3-glucanases, also known as laminarinases^63^, from nine different families (Supplementary File S2). The two most abundant enzymes were putative endo-β-1,3-glucosidases (EC 3.2.1.39): a GH17 (g2064.t1) and a GH55 (g36.t1). These were also the most abundant in the fermentations with algae (Supplementary File S2) and most likely linked to the observed endo-β-1,3-glucanase activity (Fig. 5a).

The *P*. *salina* fermentations with *A*. *nodosum* diverged from the other fermentations with high percentages of AA7 glucose-oxidases, AA3-oxidoreductases and a putative gluconolactonase. Together, these enzymes constituted as much as 47% of the secretome (Fig. 7b). The essential activities for gluconic acid production in *Aspergillus niger* were found to consist of an AA3 glucose-oxidase (EC 1.1.3.4), a catalase (EC 1.11.1.6) and a gluconolactonase (EC 3.1.1.17)^64^.

Of other noteworthy observations was a GH11 endo-β-1,4-xylanase, which was upregulated in the *F*. *serratus* fermentation (Supplementary File S2). This enzyme was most likely responsible for the activity observed on arabinoxylan and xylan (Fig. 5a). Additionally, two putative pectate lyases (EC 4.2.2.2), a PL3 and a PL1 were observed solely in the fermentations with algae (Supplementary file S2).

Proteins upregulated in the fermentations with algae other than CAZymes included abundant proteases/peptidases, a highly abundant lipase and a catalase (Supplementary File S2). One of the most abundant single proteins in all the *P*. *salina* fermentations was a 193aa long unknown protein with homology to allergen-like proteins in other fungi (g5788.t1) (Fig. 7).

## Discussion

The annotation analysis of the *P*. *salina* genome revealed a repertoire of putative enzymes, which theoretically degrade the majority of the polysaccharides in brown algae, including alginate. The *P*. *salina* genome harbored 150–250 fewer CAZymes and 60–130 fewer sugar transporters compared to its close terrestrial relatives (Table 1). However, in spite of the lower numbers of CAZymes, *P*. *salina* still harbors CAZymes relevant for degradation of most of the major types of terrestrial polysaccharides, e.g. cellulases, pectinases, hemicellulases and starch-degrading enzymes.

The proteomic and genomic analysis of the CAZymes of *P*. *salina* strongly suggests that presence of the PL7 alginate lyases is one of the most significant adaptations of *P*. *salina* to thrive on brown macro-algae. The absence of alginate lyase families in the terrestrial relatives, the absence of introns in the genes and the phylogenetic placement among bacterial sequences could indicate possible horizontal gene transfers from marine bacteria, granting *P*. *salina* the ability to degrade alginate. This hypothesis however, is difficult to confirm and the GC content analysis of the genes and the low sequence identity to all other PL7 proteins provided no further evidence. The gene loss of the PL7 genes in the terrestrial fungi is also a possibility, but even more difficult to confirm. No alginate lyases from other enzyme families were found in *P*. *salina*, thus it is reasonable to conclude that one of these genes is identical to a poly-specific β-1,4-mannuronide lyase (EC 4.2.2.3) isolated from the supernatant of a different strain, *P*. *salina* IFO32139^65^. However, this study did not produce the sequence of the protein and therefore no family classification or homology to other enzymes was provided. Our phylogenetic analysis of the PL7 sequences from *P*. *salina* indicated them to be mannuronic acid specific, which we confirmed by performing enzyme kinetics on the purified PsAlg7A, which also showed a low activity towards guluronic acids, which seems to be a common occurrence in the PL7 family^53,66^. Considering that the carbohydrate analysis of the algae used in this study showed mannuronic acid contents to be approximately two-fold higher than guluronic acid contents in all three brown algae species, it is plausible that the specificities of the enzymes would mirror this composition. To our knowledge PsAlg7A is the first recombinantly expressed and characterized PL7 from a marine fungus. The combined data from the activity assays and proteomic analysis confirmed that the PL7 proteins were abundantly present and active in the fermentations with brown algae. Our study thus indicates that *P*. *salina* is capable of degrading the alginate in the brown algae cell wall, thereby exposing other polysaccharides for enzymatic degradation. Such polysaccharides may for example be cellulose microfibrils, which *P*. *salina* appears to be able to degrade due to the numerous putative cellulases in its genome and corroborated by their active presence in the fermentation supernatants. This is not surprising considering that cellulases are quite common enzymes found in many if not all plant cell wall degrading fungi as well as in fungi not typically associated with cellulose degradation^33^. Cellulase activity and quantity were significantly higher in the algal fermentations compared to the carbon limited fermentation (Fig. 5a). Yet the presence of minor amounts of cellulase activity in the carbon limited fermentation suggests these enzymes are either constitutively expressed in small amounts or induced by starvation.

The carbon storage compounds in brown algae all have similar roles and structures in fungi. There is for example a partial structural overlap between the β-1,3-glucan found in fungal cell walls^67^ and the brown algal laminarin. The only differences between these compounds are the terminal d-mannitol residues in the algal M-series laminarin^14^. Filamentous fungi utilize β-1,3-glucanases in the synthesis, remodeling and recycling of the β-1,3-glucan in their cell wall, but also in connection with mycoparatism^68^. Our data indicates that the same putative β-1,3-glucan active enzyme, which is expressed by *P*. *salina* when carbon limited, is significantly upregulated when *P*. *salina* grows on brown algae.

Mannitol is an abundant sugar found in both fungi^60^ and brown algae^14^ and mostly related to carbon storage and stress tolerance. *P*. *salina* has been shown to utilize mannitol as sole carbon source^8^. This indicates that *P*. *salina* can metabolize extracellular sources of mannitol. We found genes belonging to both the mannitol I and II degradation pathways, which indicates that *P*. *salina* is capable of both synthesis and metabolization. Similarly, trehalose is found in both fungi^69^ and brown algae^14^. Fungi are known to utilize both cytosolic and extracellular sources of trehalose^70^, and our data suggests that this is the case in *P*. *salina* as well.

The numerous catalases and peroxidases may allow *P*. *salina* to cope with defense-related oxidative bursts in the form of hydrogen peroxide from brown algae. Such responses have been observed in *Laminaria digitata* where oligo-guluronates from alginate degradation was observed to elicit oxidative defenses in the algae^71^. The VHPOs may play a similar role^72^ during attack by the fungus by remobilizing halides from the algae or playing a role in hyphal invasion of the algal cell wall, as has been hypothesized for the many terrestrial fungi where a similar enzyme has been found^73^. Reactive oxygen species (ROS), including hydrogen peroxide, play several critical roles in terrestrial plants during plant-fungal interactions^74^ and fungi have numerous coping mechanisms in place for dealing with ROS^75,76^. Recently, it has been observed that LPMOs are effectively activated by hydrogen peroxide prior to the degradation of substrates like cellulose^77^. The numerous AA9 LPMOs found in the *P*. *salina* genome and in the algal fermentations suggest that they play an important role in the degradation of the brown algal cell. It is possible that the fungal LPMOs in synergy with peroxidases could provide an ingenious way to use the algal/plant defense against itself while speeding up the degradation of polysaccharides like cellulose and hemicellulose. Extensive additional experiments would need to be performed to confirm these hypotheses. Unfortunately this stretches beyond the scope of this study.

The identification of a putative PL8 chondroitin AC lyase gene in *P*. *salina* without any equivalent genes in the compared terrestrial fungi and the fact that this enzyme was highly abundant in the algal fermentations raises interesting questions regarding the role and substrate specificity of the lyase. Chondroitin sulfate belongs to the glycosaminoglycan (GAG) family of linear heteropolysaccharides attached covalently to proteins forming proteoglycans^78^. These types of proteoglycans are almost exclusively associated with animal tissues and cells^79^ and to our knowledge have never been found in algae or fungi. Two putative PL8 proteins from *Trametes versicolor* F21a (Basidiomycota) were previously found by proteomic analysis when the fungus fermented microalga, one of which significantly upregulated^80^.

Aside from the numerous β-glucan active enzymes, the *P*. *salina* genome like that of *S*. *lycopersici* contains abundant hemicellulolytic and pectinolytic enzymes. As many as 36 putative pectinase genes were annotated in the genome suggesting that *P*. *salina* effectively degrades pectin. The shared sequence identities with *S*. *lycopersici* pectinases, like many of the other CAZymes in the *P*. *salina* genome, indicate commonalities between marine and terrestrial sources of pectin. Pectinases indeed seem to be a common occurrence in marine ascomycetes^6,81^ and they can even be induced by terrestrial sources of pectin^82^. In the case of marine bacteria, various horizontal gene transfers of whole pectinolytic loci from terrestrial bacteria have been observed, which might indicate an evolutionary pressure towards the metabolization of marine pectin, said to stem from seagrasses and marine diatoms^83,84^. The two putative pectate lyases found solely in the *P*. *salina* fermentations with brown algae raises an interesting question regarding their substrate specificities. Similarly, in the fermentation with *F*. *serratus* as substrate, the observation of endo-xylanase activity on xylan and arabinoxylan linked to the presence of a GH11 endo-xylanase is slightly perplexing. The proposed model of the brown algal cell wall incorporates short-chained hemi-cellulose molecules^85^. However, to our knowledge no polysaccharide with a xylan backbone has been identified in brown algae. Like the pectinases, the large number of annotated hemicellulases in the *P*. *salina* genome is most likely relevant for seagrasses^86^ and various terrestrial sources washed out to sea.

Generally, the enzymatic capabilities of *P*. *salina* suggest that this fungus is capable of degrading most components of brown algae, seagrasses and terrestrial plant material, which is indicative of a broad and saprobic lifestyle with plant pathogenic traits. Based on the high percentage of shared CAZymes with its plant-pathogenic family members and the acquired alginate lyases, it seems plausible that *P*. *salina* belongs to the proposed group of marine fungi that were secondary colonizers from the terrestrial to the marine environment^6,87^.

This study provides detailed insights into the adaptations and brown algae enzymatic degradation pattern in a marine fungus. Our genome analysis indicates that the repertoire of enzymes found in *P*. *salina* is highly similar to that of terrestrial saprobic and plant pathogenic fungi from the same family. With this enzymatic repertoire and an acquired ability to degrade alginate, *P*. *salina* is capable of degradation and metabolization of most major polysaccharides from brown algae.

## Supplementary information

Supplementary information

Supplementary File S1

Supplementary File S2

Supplementary File S3

Supplementary File S4

## Data Availability

The draft genome and transcriptome assemblies, along with the cDNA paired-end reads were submitted to the European Nucleotide Archive (ENA) (www.ebi.ac.uk/ena) under the study accession number PRJEB30354. The mass spectrometry proteomics data have been deposited to the ProteomeXchange Consortium via the PRIDE partner repository with the dataset identifier PXD012152 (www.proteomexchange.org/). All other data generated or analyzed during this study are included in this published article and its Supplementary Information files.
