# Supplementary material for: Proteomic enzyme analysis of the marine fungus Paradendryphiella salina reveals alginate lyase as a minimal adaptation strategy for brown algae degradation
Source: Sci Rep. 2019 Aug 26;9:12338. doi: 10.1038/s41598-019-48823-9 (PMC6710412; doi:10.1038/s41598-019-48823-9)
Supplement: Supplementary file 1 — Supplementary information [file 41598_2019_48823_MOESM1_ESM.pdf]

Supplementary Information

Proteomic enzyme analysis of the marine fungus *Paradendryphiella salina* reveals alginate lyase as a minimal adaptation strategy for brown algae degradation

Bo Pilgaard[1]*, Casper Wilkens[1], Florian-Alexander Herbst[2], Marlene Vuillemin[1], Nanna Rhein-Knudsen[1], Anne S. Meyer[1] and Lene Lange[3]

[1]Enzyme Technology, Department of Biotechnology and Biomedicine, Technical University of Denmark

[2]Center for Microbial Communities, Department of Chemistry and Bioscience Aalborg University, Denmark

[3]BioEconomy, Research & Advisory, Denmark

*Corresponding author:

Bo Pilgaard, Søltofts Plads, Building 221,DK-2800, Kongens Lyngby, Denmark, bpil@dtu.dk

Email of other authors:

CW - cwil@dtu.dk

FH - fah@bio.aau.dk

MV - mavu@dtu.dk

NK - nark@dtu.dk

AM - asme@dtu.dk

LL - lene.lange2@gmail.com

Supplementary figures and tables

	#	Protein in supernatants	Volume used for TCA	Protein* in 100ul resolubilized GdHcl	Factor used for MS data normalization
Carbon starved	G1	0.00068	40ml	0.271	0.011
	G2	0.00087	40ml	0.349	0.014
	G3	0.00118	40ml	0.472	0.019
	G4	0.0011	40ml	0.439	0.017
	G5	0.00052	40ml	0.208	0.008
A. nodosum	A1	n.a	n.a	n.a	n.a
	A2	0.01137	2ml	0.227	0.180
	A3	0.01372	2ml	0.274	0.218
	A4	0.01058	2ml	0.212	0.168
	A5	0.01263	2ml	0.253	0.200
F. serratus	F1	0.03158	2ml	0.632	0.501
	F2	0.03439	2ml	0.688	0.545
	F3	0.03297	2ml	0.659	0.523
	F4	0.04017	2ml	0.803	0.637
	F5	0.03466	2ml	0.693	0.549
S. latissima	S1	0.05178	2ml	1.036	0.821
	S2	0.0461	2ml	0.922	0.731
	S3	0.0428	2ml	0.856	0.679
	S4	0.06307	2ml	1.261	1.000
	S5	0.04419	2ml	0.884	0.701

*BCA measurement in mg/ml

Table S1. Protein concentrations in fermentation supernatants. Protein concentrations determined by the Pierce BCA Protein Assay Kit. Includes the calculated dilution factors for each sample used for normalizing the LC-MS/MS iBAQ values.

AZCL substrate	Polysaccharide description	Endo-enzyme activity listed by supplier (Megazyme)
Dextran	Dextran	Dextranase
Amylose	Amylose	α-amylase
Curdlan	Liniar β-1,3-glucan with very few 1,6 internal branch points	β-1,3-glucanase
Pachyman	Branched β-1,3-glucan with a 1,3 branch point on every 50 glucose and few 1,6 internal branch point every five residue	β-1,3-glucanase
β-glucan (barley)	1,3-1,4 mixed linkage β-glucan	β-1,3-1,4 -glucanase
Galactan (potato)	Liniar β-1,4-galactan	β-1,4-galactanase
HE-cellulose	Amorphous cellulose	β-1,4-glucanase
Galactomannan (Carob)	β-1,4-mannan with galactose substitutions	β-1,4-mannanase
ArabinoXylan (wheat)	Liniar β-1,4-xylan with arabinose substitions	β-1,4-xylanase
Xylan (birch)	Linar β-1,4-xylan	β-1,4-xylanase
Xyloglucan (tamarin)	β-1,4-glucan backbone with α-D-xylopyranose substitutions	β-1,4-glucanase
Casein	Casein	Protease

Table S2. The selected AZurine-Cross-Linked substrates, structural description and supposed enzyme activity as described by the supplier.

Name	Gene
PsAlg7A	TACACTGCTCCATCTACTGAATCTAAGTTTACTGAGGTTTTGTCTAAGGCTAAATTGCAATATCCTACTTCTACTACTGTTGCTTTCGCTGATGATTTGTTGGATGGTTACGCTGCTTCTTACTTCTATTTGACTTCTGATTTGTACATGCAATTCCAAGTTGCTGGTTCTTCTCAAAGATCTGAATTGAGAGAAATGGAGACTTCTGGAGATGAGGCTGCTTGGGATTGTACTGGTTCTACTGCTCATGTTGCTTCTGCTCAAATTGCTATTCCAGTTCAAGAAGATGGTATTGAAGAGGTTACTATTTTGCAAGTTCACGATTCTGATGTTACTCCTGTTTTGAGAATCTCTTGGGTTTCTTCTATCACTATCGATGGTGTTACTTCTGAAGATGTTGTTTTGGCTACTATCAGAAACGGTATCGATGATTCTACTGCTACTAAGACTGTTTTGCAAGCTCATACTACTTCTAGAACTGAGTTTAATATCAACGTTCAAAACTCTAAGTTGTCTATCACTGTTGATGGTACTACTGAATTGGATGAGGCTGATATCTCTCAATTCGATGGTTCTACTTGTTACTTCAAGGCTGGTGCTTATAACAATAACCCAACTGATACTTCTGCTAACGCTAGAATTAAAATGTACGAATTGGAGTGG

Table S3. Nucleotide sequence encoding PsAlg7A. Codon optimized for *P. pastoris*.

Type	Number of reads	Sample Yield (in MB)	Average Quality scores (Phred)	Total reads	Mapped reads
Genome	14426983	3418	38	28853966	27826167
Transcriptome	9899844	2441	38	19799688	18962918

Type	Mapped reads in pairs	Average coverage	Insert size median	Scaffolds	Sum (bp)
Genome	27457561	120,43	303	1818	27.384.128
Transcriptome	18588240	61,12	201	21394	38280753

Type	GC (%)	Max scaffold size	Min. scaffold size	Average scaffold size	N25
Genome	52,13	139	302	15	54
Transcriptome	53	17552	201	1789	4453

Type	N50	N75	Gaps	Size of gaps
Genome	33	17	108	241
Transcriptome	2792	1643	0	0

Table S4. Sequencing and assembly statistics from the *P. salina* genome and transcriptome assemblies.

Query	Description	Max score	Total score	Query cover	E-value	Identity	Accession
MH873443.1	Stemphylium lycopersici strain CIDEFI 213 scaffold_250	2145	2145	100%	0	98%	QGDH01000250.1
	Stemphylium lycopersici strain CIDEFI 212 scaffold_311	2145	2145	100%	0	98%	QGDG01000311.1
	Curvularia sp. IFB-Z10 scaffold_33	1901	1901	100%	0	94%	JPSZ01000033.1
	Bipolaris maydis C5 COCHEscaffold_9_Cont22	1881	1881	100%	0	94%	AIDY01000022.1
	Alternaria brassicicola strain Abra43 tig00000030_pilon	1875	3750	100%	0	94%	PHFN01000024.1
	Alternaria brassicicola strain Abra43 tig00000029_pilon	1862	2574	100%	0	94%	PHFN01000023.1
	Bipolaris oryzae isolate TG12bL2 contig_45	1862	1862	100%	0	94%	LNFW01000034.1
	Alternaria sp. MG1 NODE_21	1858	1858	100%	0	94%	QPFE01000018.1
	Alternaria alternata strain B2a contig_50	1858	1858	100%	0	94%	LSHC01000019.1
	Alternaria alternata strain Z7 contig089-7934	1858	3717	100%	0	94%	LPVP01000083.1

Table S5. Top hits from the BLASTn analysis of the consensus ribosomal nucleotide sequence from *P. salina* (accession MH873443.1).

Family	*P. salina*	*S. lycopersici*	*B. maydis*	*A. alternate*
GH1	3	3	3	3
GH2	5	8	7	7
GH3	11	15	17	18
GH5	15	20	17	21
GH6	2	3	3	3
GH7	4	4	6	4
GH10	3	4	5	7
GH11	3	5	5	5
GH12	2	3	3	5
GH13	7	7	7	7
GH15	2	2	2	2
GH16	18	18	17	18
GH17	6	6	6	8
GH18	10	12	16	12
GH20	2	3	3	3
GH26	0	1	1	1
GH27	1	4	3	3
GH28	6	7	3	8
GH29	0	0	1	0
GH30	2	2	2	3
GH31	5	9	9	10
GH32	3	3	3	3
GH33	0	0	1	1
GH35	2	6	4	6
GH36	1	1	1	1
GH37	2	2	2	2
GH38	1	1	1	1
GH43	9	20	18	20
GH45	2	2	3	3
GH47	10	11	11	10
GH51	2	2	2	2
GH53	0	1	1	1
GH54	0	1	1	1
GH55	3	4	3	5
GH62	3	2	2	2
GH63	3	3	3	3
GH64	1	1	1	1
GH65	1	1	1	1
GH67	1	1	1	1
GH71	0	1	1	1
GH72	6	6	7	6
GH74	3	4	4	4
GH76	8	10	9	10
GH78	2	5	3	7
GH79	1	3	3	3
GH81	3	2	2	2
GH85	1	1	1	1
GH88	0	1	1	2
GH92	1	6	5	5
GH93	1	2	4	2
GH94	1	1	1	1
GH95	2	2	2	2
GH105	3	3	4	4
GH106	0	0	1	0
GH109	9	11	8	16
GH113	0	1	0	0
GH114	1	1	1	2
GH115	2	2	3	5
GH125	3	3	3	3
GH127	0	1	0	0
GH128	5	5	6	6
GH131	3	3	5	4
GH132	1	1	1	1
GH134	0	1	0	0
GH135	1	2	2	2
GH141	0	0	1	0
GH142	0	1	0	1
GH145	1	1	1	1
Subtotal	209	278	274	303
Total	478	616	607	699
Diff		138	129	221
Diff %		22	21	32
AA	86	136	141	167
CE	55	80	77	95
PL	22	23	16	26
GH	209	278	274	303
GT	82	99	99	108

Family	*P. salina*	*S. lycopersici*	*B. maydis*	*A. alternate*
AA1	7	7	8	8
AA2	5	7	10	9
AA3	13	30	32	41
AA4	2	3	4	4
AA5	5	5	5	5
AA6	1	1	1	1
AA7	20	46	43	57
AA8	1	0	1	1
AA9	25	28	24	32
AA10	0	0	0	0
AA11	4	5	9	5
AA12	1	3	3	3
AA13	1	1	1	1
Subtotal	85	136	141	167
CE1	18	28	31	37
CE2	1	1	0	1
CE3	2	4	4	7
CE4	11	14	14	16
CE5	8	15	13	14
CE7	0	1	0	0
CE8	5	7	3	7
CE9	2	2	3	2
CE12	4	3	4	5
CE14	1	0	1	1
CE15	1	2	1	2
CE16	2	3	3	3
Subtotal	55	80	77	95
PL1	7	10	6	11
PL3	5	6	5	7
PL4	5	4	4	4
PL7	3	0	0	0
PL8	1	0	0	0
PL9	0	1	0	1
PL11	0	0	0	1
PL22	0	1	0	1
PL26	1	1	1	1
Subtotal	22	23	16	26
GT1	5	9	6	9
GT2	12	15	16	14
GT3	1	1	1	1
GT4	5	5	5	6
GT8	6	5	8	6
GT15	3	4	4	4
GT20	3	3	3	3
GT21	1	2	1	2
GT22	4	3	4	4
GT23	0	0	1	0
GT24	1	1	1	1
GT25	1	2	2	4
GT28	0	0	1	0
GT31	4	4	3	7
GT32	4	7	8	9
GT33	1	1	1	1
GT34	8	10	9	9
GT35	1	1	1	1
GT39	3	3	3	3
GT48	1	1	1	1
GT50	1	1	1	1
GT54	1	1	0	0
GT55	0	0	0	1
GT57	3	2	3	3
GT58	1	1	1	1
GT59	1	0	1	1
GT62	3	3	3	3
GT66	1	1	1	1
GT69	3	4	2	3
GT71	1	1	1	2
GT76	0	1	1	1
GT90	3	7	6	6
Subtotal	82	99	99	108

Table S6. HMMer analysis using dbCAN models of the fungal genomes used in this study. This data was used for the VENN analysis.

	Description	Max score	Total score	Query cover	E value	Identity	Accession
g8132.t1	polysaccharide lyase family 7 protein [Nitratiruptor sp. SB155-2]	89	89	90%	1.00E-17	30%	WP_012081562.1
	polysaccharide lyase family 7 protein [Gayadomonas joobiniege]	80.9	80.9	90%	2.00E-14	29%	WP_017446946.1
	polysaccharide lyase family 7 protein [Aquimarina pacifica]	79.3	79.3	91%	2.00E-13	31%	WP_025741611.1
	hypothetical protein [Aquimarina aggregata]	74.7	74.7	89%	9.00E-12	28%	WP_066313446.1
	polysaccharide lyase family 7 protein [Actinoplanes globisporus]	67.4	67.4	65%	1.00E-09	32%	WP_020512297.1
	T9SS C-terminal target domain-containing protein [Lewinella cohaerens]	64.7	64.7	48%	3.00E-08	34%	WP_020534342.1
	hypothetical protein BC351_05040 [Paenibacillus ferrarius]	62	62	78%	1.00E-07	28%	OPH57857.1
	putative alginate lyase AlyVOB [Alcanivorax hongdengensis A-11-3]	62.4	62.4	88%	1.00E-07	26%	EKF75036.1
	MULTISPECIES: polysaccharide lyase family 7 protein [Streptomyces]	62	62	98%	1.00E-07	28%	WP_019072329.1
	polysaccharide lyase family 7 protein [Paenibacillus ferrarius]	62	62	78%	1.00E-07	28%	WP_079413516.1
g6933.t1	Alginate lyase 2 domain protein [Rhodopirellula sp. SWK7]	96.3	96.3	92%	2.00E-20	30%	EMI43849.1
	polysaccharide lyase family 7 protein [Rhodopirellula sp. SWK7]	96.7	96.7	92%	3.00E-20	30%	WP_052329521.1
	hypothetical protein BU14_1709s0001 [Porphyra umbilicalis]	93.6	93.6	80%	2.00E-19	32%	OSX69237.1
	polysaccharide lyase family 7 protein [Vibrio hyugaensis]	89.4	89.4	96%	1.00E-17	28%	WP_052437450.1
	polysaccharide lyase family 7 protein [Caminibacter mediatlanticus]	82	82	90%	4.00E-15	29%	WP_007473671.1
	hypothetical protein [Alcanivorax nanhaiticus]	73.9	73.9	81%	7.00E-12	27%	WP_035229412.1
	hypothetical protein [Alcanivorax sp. P2S70]	68.2	68.2	69%	7.00E-10	28%	WP_022986244.1
	putative alginate lyase AlyVOB [Alcanivorax hongdengensis A-11-3]	66.6	66.6	81%	2.00E-09	27%	EKF75036.1
	MULTISPECIES: hypothetical protein [Lebetimonas]	65.1	65.1	57%	3.00E-09	31%	WP_024788376.1
	hypothetical protein [Alcanivorax hongdengensis]	66.6	66.6	81%	3.00E-09	27%	WP_083851681.1
g8867.t1	Alginate lyase 2 domain protein [Rhodopirellula sp. SWK7]	101	101	94%	3.00E-22	32%	EMI43849.1
	polysaccharide lyase family 7 protein [Rhodopirellula sp. SWK7]	100	100	96%	6.00E-22	33%	WP_052329521.1
	polysaccharide lyase family 7 protein [Psychromonas sp. SP041]	85.1	85.1	86%	7.00E-16	31%	WP_081695320.1
	hypothetical protein [Aquimarina aggregata]	78.6	78.6	87%	4.00E-13	28%	WP_066313446.1
	polysaccharide lyase family 7 protein [Hippea jasoniae]	69.3	69.3	94%	2.00E-10	27%	WP_051904545.1
	polysaccharide lyase family 7 protein [Aquimarina pacifica]	67.8	67.8	91%	2.00E-09	26%	WP_025741611.1
	hypothetical protein [Psychromonas sp. SP041]	57	57	96%	8.00E-06	25%	WP_025563003.1
	putative alginate lyase AlyVOB [Alcanivorax hongdengensis A-11-3]	49.3	49.3	59%	0.002	29%	EKF75036.1
	hypothetical protein [Alcanivorax hongdengensis]	49.3	49.3	59%	0.003	29%	WP_083851681.1
	hypothetical protein [Rhizobiales bacterium]	49.3	49.3	58%	0.003	30%	WP_113106616.1

Table S7. Top hits from the BLASTp analysis of the putative PL7 protein sequences found in the *P. salina* genome.

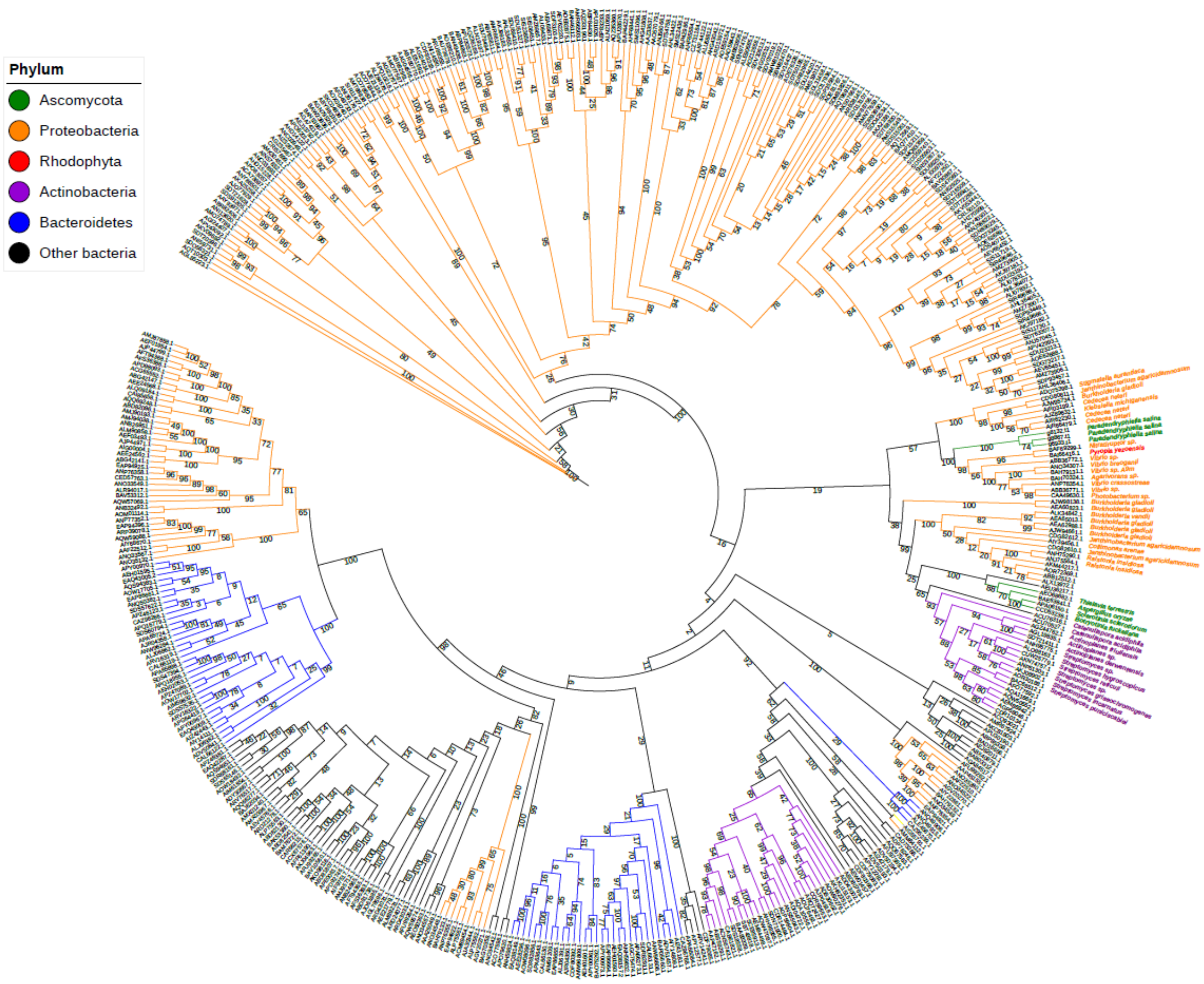

Figure S1. Maximum-likelihood phylogenetic tree (including bootstrap values and accession numbers) of all the PL7 sequences listed in the CAZy database. Only the catalytic domains predicted by dbCAN were used for the multiple alignment. The colors of branches and organism names represents phylae. The clades selected for figure 4 are highlighted by organism names.

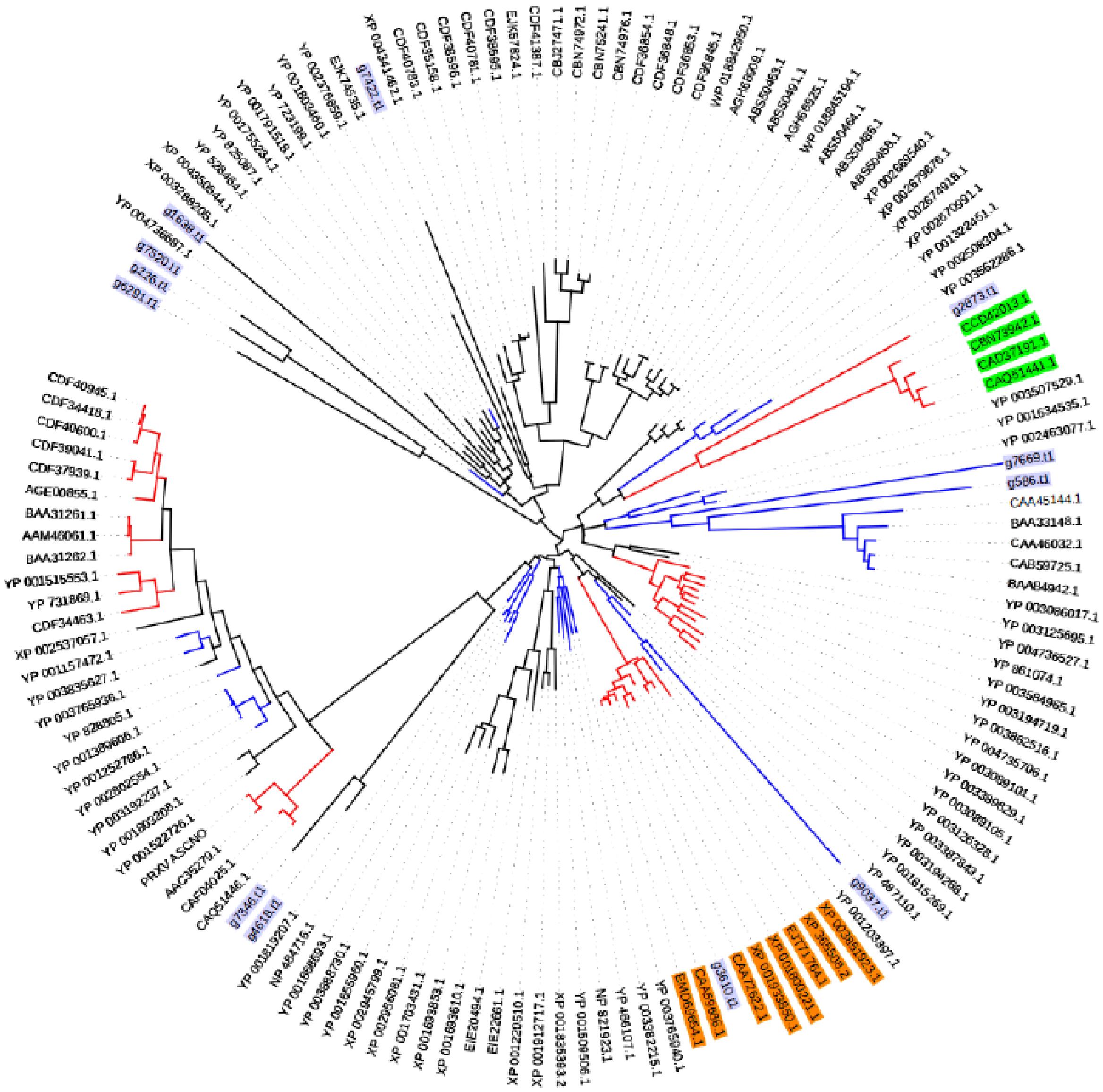

Figure S2. Maximum likelihood phylogeny of PAP2 containing sequences from *P. salina* aligned with known Vanadium-Halo-PerOxidases (VHPO) and acid phosphatases. Blue colored accession numbers indicate *P. salina* sequences; orange accession numbers indicate fungal vanadium-dependent chloroperoxidases and bright green indicate vanadium-dependent bromoperoxidases from macro algae. Blue branches indicate acid phosphatases and red branches VHPOs.

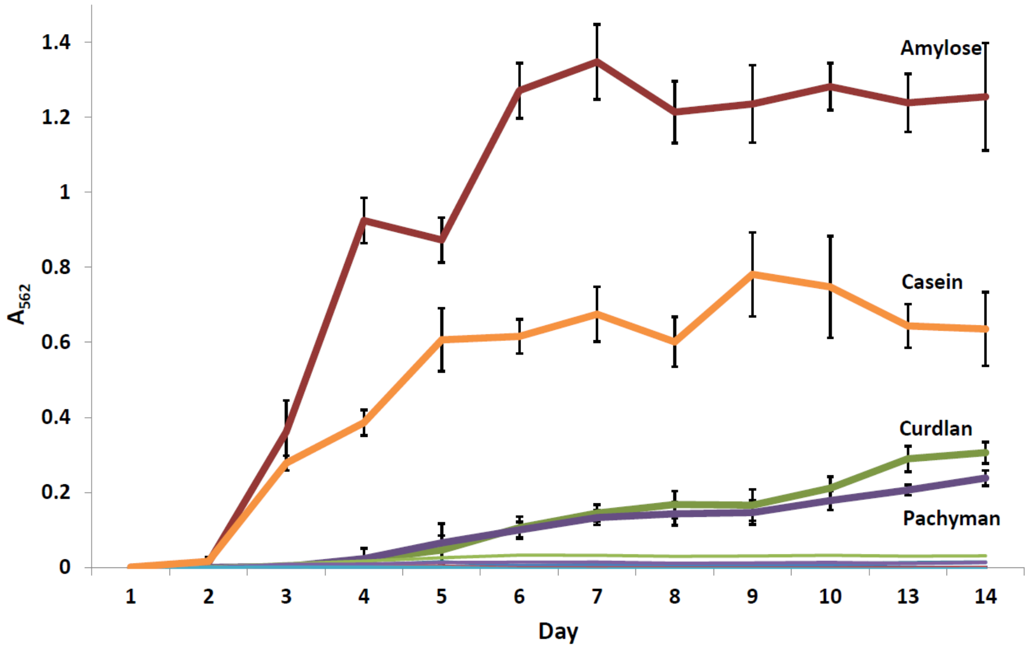

Figure S3. 14-day time-course experiment following the carbon limited *P. salina* fermentations. All AZCL substrates were tested for activity.

Fucoidan from *Fucus vesiculocus*

Incubation: 48h, 35C

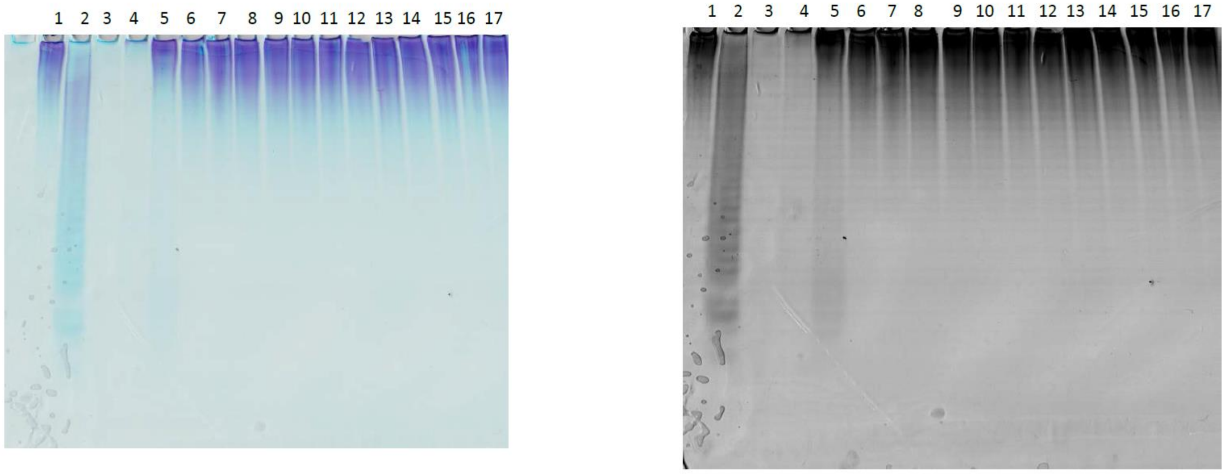

	Controls					Reactions											
Lane	1	2	3	4	5	6	7	8	9	10	11	12	13	14	15	16	17
Description	Substrate	Degraded substrate	F5 supernatant	S5 supernatant	A5 supernatant	G3	G4	G5	F3	F4	F5	S3	S4	S5	A3	A4	A5

Figure S4. C-page results from enzymatic reactions using three out of five *P. salina* supernatants from day 14 on fucoidan from *F. versiculocus*.

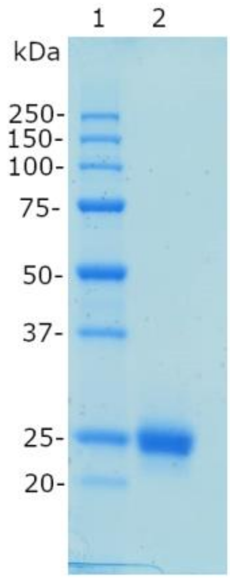

Figure S5. 12% SDS-PAGE gel of the purified PsAlg7A.
